# Comparison of the Effect of Ranibizumab and Aflibercept on Changes in Macular Choroidal Thickness in Patients Treated for Diabetic Macular Edema

**DOI:** 10.1155/2020/5708354

**Published:** 2020-08-11

**Authors:** Valérie Sarda, Pauline Eymard, Linda Hrarat, Franck Fajnkuchen, Audrey Giocanti-Aurégan

**Affiliations:** ^1^Ophthalmology Department, Hôpital Avicenne, 125 Rue de Stalingrad, Bobigny 93000, France; ^2^Centre Ophtalmologique Saint Paul Bastille, 19 Rue Saint Antoine, Paris 75004, France; ^3^Centre D'imagerie et de Laser, Paris 75015, France

## Abstract

**Purpose:**

The aim of this study was to assess the effect of intravitreal injections (IVI) of ranibizumab and aflibercept on the choroidal thickness (CT) in patients with treatment-naive diabetic macular edema (DME) before and after monthly IVI. *Patients and Methods*. Prospective monocenter study. Inclusion criteria were treatment-naive DME eyes without concomitant panretinal photocoagulation, associated with a decrease in best-corrected visual acuity ≤75 letters on the Early Treatment Diabetic Retinopathy Study (ETDRS) scale. DME was defined by a central retinal thickness ≥300 *μ*m on swept-source OCT (Triton DRI OCT, Topcon Corporation, Itabashi, Japan). Patients received 5 IVI of ranibizumab or aflibercept. The primary endpoint was the change in the central subfield CT (CSCT) between inclusion (M0) and 1 month after the fifth IVI (M5). The secondary endpoint was the CT changes between M0 and M5 in other locations of the macular ETDRS grid.

**Results:**

Twenty-four eyes of 24 patients with a mean age of 61.1 years were included. Eleven and 13 patients were, respectively, treated with ranibizumab and aflibercept, and 86.4% had type 2 diabetes. The overall CSCT decreased significantly by −12 *μ*m between M0 and M5 (231.7 *μ*m at M0 and 219.7 *μ*m at M5) (*p*=0.03). It decreased by −15.2 *μ*m (*p*=0.02) in the aflibercept group (206.9 *μ*m at M0 and 191.7 *μ*m at M5) and by −7.3 *μ*m (*p*=0.4) in the ranibizumab group (267.5 *μ*m at M0 and 260.2 *μ*m at M5). The CSCT decreased by −4.9 *μ*m in noninjected contralateral eyes (242.3 *μ*m at M0 and 237.4 *μ*m at M5). CT changes between M0 and M5 in the superior, temporal, inferior, and nasal macular inner ring were significant in the aflibercept group but not in the ranibizumab and control groups.

**Conclusion:**

In DME patients, the CSCT decreases after 5 IVI of anti-VEGF, especially after aflibercept treatment.

## 1. Introduction

The first-line therapy of central diabetic macular edema (DME) with decreased visual acuity is usually based on intravitreal injections (IVI) of anti-VEGF [1] that have proved their efficacy on visual impairment reduction [2]. Ranibizumab and aflibercept, the two anti-VEGFs currently used in France, have been shown to be effective in reducing DME and improving vision in eyes with DME [3, 4]. Anti-VEGFs have not only an effect on the retinal vasculature but also on the choroidal thickness (CT). Also, the choroidal vasculature is known to be involved in diabetes. Changes in the CT and choroidal blood flow have been reported in eyes with DME compared to healthy eyes [5]. In addition, in neovascular age-related macular degeneration (nAMD), studies have shown a choroidal thinning under anti-VEGF, especially with aflibercept [6]. The aim of this study was to assess the effect of intravitreal injections (IVI) of ranibizumab and aflibercept on the CT in treatment-naive DME patients after a loading phase of 5 monthly IVI.

## 2. Material and Methods

A prospective, monocenter study was conducted in the ophthalmology department of the Avicenne Hospital, Bobigny (Seine Saint Denis), France. This study was conducted in accordance with the tenets of the Declaration of Helsinki, and an informed consent was obtained from all subjects. Approval was obtained from the France Macula Federation ethics committee.

Consecutive patients with unilateral macular edema were included, and the contralateral eye was used as the healthy control eye.

Inclusion criteria were age >18 years, type 1 or type 2 diabetes, DME defined by a central retinal thickness (CRT) ≥300 microns using swept-source OCT (DRI OCT Triton, Topcon corporation, Itabashi, Japan), and a visual acuity ≤75 letters on the Early Treatment Diabetic Retinopathy Study (ETDRS) scale.

All patients were naive of treatment, without concomitant panretinal photocoagulation (PRP), and underwent an initial loading phase of 5 monthly IVI with 0.5 mg of ranibizumab or 2 mg of aflibercept. Treatment assignation was randomized 1 : 1. All patients received treatment in only one eye. The contralateral eye did not show any macular edema and was included in a healthy control group without IVI when no exclusion criteria were present.

Exclusion criteria were active proliferative diabetic retinopathy (DR), intravitreous hemorrhage or tractional retinal detachment, ischemic maculopathy (defined by a 2-time enlargement of the foveal avascular zone assessed by fluorescein angiography), or any other ocular pathology contributing to the decrease in visual acuity.

All patients underwent a complete ophthalmological examination including the best-corrected visual acuity (BCVA) on the ETDRS scale, a slit-lamp examination, a noncontact fundus examination (Superfield, Volk), ultrawide field angiography (California, Optos PLC), and macular OCT with CRT and the CT measurement (DRI OCT Triton, Topcon Corporation, Itabashi, Japan).

Fluorescein angiography was performed during the initial examination to exclude ischemic maculopathy, to assess DR, and to rule out another retinal disease.

Patients underwent a complete ophthalmological examination one month after the fifth IVI including BCVA on the ETDRS scale, a slit-lamp examination, a noncontact fundus examination (Superfield, Volk), ultrawide field retinophotography, and macular OCT with CRT and the CT measurement (DRI OCT Triton, Topcon Corporation, Itabashi, Japan).

**Figure 1:**
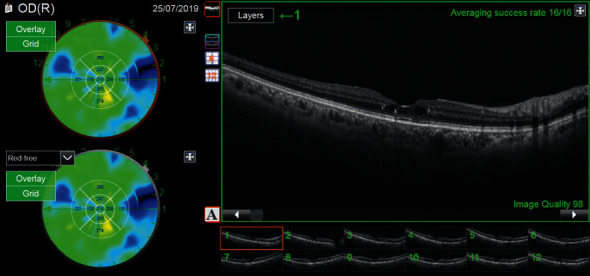
Choroidal mapping of a diabetic patient eye after DME treatment.

The primary endpoint was the change in the central subfield CT (CSCT) for each patient between M0 and 1 month after the fifth IVI (M5). This measurement was an automatic measurement of the choroid with Topcon swept-source OCT (automatic mapping). This measurement was only made in the afternoon OCT session between 2 and 5 pm (Figure 1) in order to avoid the diurnal change in the CT.

The secondary endpoint was the CT changes between M0 and M5 in other locations of the ETDRS macular inner ring.

Results are expressed as the mean ± standard deviation. Statistical analyzes were performed using the Wilcoxon nonparametric matched-pairs test for intrapatient comparisons of the paired CT (M5 versus M0) and the Mann–Whitney test for comparison between unpaired values, using the GraphPad Software Prism 7.0. Statistical significance was set at *p* < 0.05.

## 3. Results

Twenty-four eyes of 24 patients with vision loss due to DME were included in the study.

Patient characteristics (presented in Table 1) were a mean age of 61.1 years and a sex ratio of 2.14. Most patients had type 2 diabetes (86.4%).

Among these 24 patients, 13 were randomized to the aflibercept group, and 11 were randomized to the ranibizumab group. Two patients (2/13) in the ranibizumab group did not complete the loading phase and were excluded. All patients had an untreated healthy contralateral control eye, but 9 developed a DME during the follow-up or underwent PRP and were excluded. The control group included 15 eyes of 15 patients. Visual acuity was 65.4 ETDRS letters at baseline and 75.5 ETDRS letters at M5 in patients treated with anti-VEGF. Thus, the mean visual gain was +10.1 ETDRS letters (*p* < 0.0001).

The mean CRT was 359.7 microns at baseline and 294.6 microns at M5, corresponding to a mean decrease in CRT of −65.1 microns (*p*=0.008).

### 3.1. Primary Endpoint

In all patients, the mean CT was 231.7 microns at baseline and 219.7 microns at M5 (*p*=0.03), corresponding to a mean decrease in the CSCT under anti-VEGF treatment by −12 microns between M0 and M5. In the aflibercept group (*n* = 11), the mean central CT was 206.9 microns at baseline and 191.7 microns at M5 (*p*=0.02), corresponding to a mean decrease in the CSCT by −15.2 microns. In the ranibizumab group (*n* = 9), the mean CSCT was 267.5 microns at baseline and 260.2 microns at M5 (*p*=0.4), corresponding to a mean decrease in the CSCT by −7.3 microns. The mean CSCT at baseline was 206.9 *μ*m in the aflibercept group and 267.5 *μ*m in the ranibizumab group; after the Mann–Whitney test, the *p* value was not significant (*p*=0.16). Medians were 234 *μ*m in the aflibercept group and 270 *μ*m in the ranibizumab group.

In the healthy control eyes (*n* = 15), the mean CSCT was 242.3 microns at baseline (238 *μ*m in the aflibercept group and 245 *μ*m in the ranibizumab group) and 237.4 microns at M5 (227 *μ*m in the aflibercept group and 245 *μ*m in the ranibizumab group) (*p*=0.34), corresponding to a mean decrease in the CSCT by −4.9 microns.

There was a trend towards a thinner choroid at baseline in DME eyes (231.7 microns) compared to eyes without DME (242.3 microns), but the difference did not reach significance (*p*=0.84).

### 3.2. Secondary Endpoint

The mean CT in the superior, temporal, inferior, and nasal parts of the ETDRS inner ring is presented in Table 2 for the aflibercept, ranibizumab, and control groups. All CT changes between M0 and M5 were significant in the aflibercept group while none was significant in the other groups.

## 4. Discussion

Our study showed that, in DME eyes, the CT decreased under treatment with anti-VEGF, and this decrease was more marked under aflibercept.

Several studies have shown changes in the CSCT in patients with retinal complications secondary to diabetes. Most studies have found a decrease in the CSCT in DR, DME, and after PRP. This finding is consistent with the known histological signs such as the narrowing of choroidal arterioles and capillary dropout in choriocapillaries in DR patients [7, 8]. However, surprisingly, some studies have not reported such a decrease and have even found an increase in the subfoveal central CT in DR patients, with a thicker choroid when DR progresses. Campos in 2017 has highlighted these discrepancies [9].

The effect of anti-VEGF on the choroid and the comparison of the effect of various anti-VEGF have already been shown in nAMD [10]. A single IVI of ranibizumab significantly affects the choroidal vasculature and the choroidal flow [11]. Anti-VEGF agents are indeed able to penetrate all retinal layers, reach the choroid, and accumulate in the wall of the choroidal vessel [12]. One of the effects of VEGF is to induce vessel dilation and to increase ocular blood flow, and this is mediated by an increased nitric oxide production [13]. Through VEGF inhibition, these agents could induce the constriction of choroidal vessels. The CT is already decreased in patients with diabetes, and the consequences of a further thinning of the choroidal vasculature secondary to anti-VEGF administration are unknown. An impaired choroidal blood flow could result in photoreceptor dysfunction and mortality. This could contribute to the greater anatomical and functional responses to anti-VEGF treatment previously reported in patients with thicker choroids [14].

In DME patients, all studies have shown that anti-VEGF injections reduce the central CT, although the clinical consequences of this reduction remain unclear. Some studies have found an association between the decrease in the central CT and the decrease in CRT and the visual gain under anti-VEGF treatment [15]. Other studies have not been able to find such an association with the visual gain [9, 16, 17]. Campos et al. [16] and Yiu et al. [17] have suggested that the CSCT was not predictive of the anatomical or functional outcomes of DME. However, in a prospective study of 20 eyes of 20 treatment-naive DME patients, a decrease in the central CT 6 months after treatment with 3 IVI of bevacizumab has been shown, and this reduction significantly correlated with a reduction in CRT and with an improvement in visual acuity [15].

In our study, we investigated the effect of anti-VEGF on the CT during a well-conducted loading phase of 5 IVI and compared the effect of two anti-VEGFs, aflibercept and ranibizumab, on the reduction of the CT in DME patients. Twenty-four eyes of 24 patients were prospectively included and randomized to one anti-VEGF. We showed a CT decrease under anti-VEGF treatment in both treated groups, but it only reached significance in the aflibercept group, for all the locations of the macular ETDRS inner ring.

In nAMD, a greater thinning of the choroid has already been demonstrated under aflibercept compared to ranibizumab [6]. In DME patients, most studies have shown a significant CT decrease, regardless of the anti-VEGF agent used, but Gharbiya et al. [6] have found, as in our series, a significant CT decrease in nAMD patients treated with aflibercept while no corresponding decrease was observed in ranibizumab-treated eyes.

The measurements performed in the ETDRS inner ring supported the assumption that aflibercept could more strongly decrease the CT than ranibizumab because, regardless of the territories explored, the CT decrease was significant in all quadrants in the aflibercept group but not in the ranibizumab group. Yun et al. [18] have already observed such a difference: they have found that IVI of aflibercept significantly decreased the subfoveal CT and that decrease was stronger than with ranibizumab, and the CT thinning observed after aflibercept injections was not limited to the subfoveal area but extended beyond the macula. Compared to ranibizumab, aflibercept more strongly reduced the CT and the number of fenestrations of the choriocapillaries in monkey eyes. One possible explanation could be the interaction between a Fc fragment and other molecules [19]. This property, which could be favorable or not, could explain the effect of aflibercept on the CT.

We have also noticed that in the control (untreated) eye of patients treated by aflibercept, there was a trend to the CSCT decrease after 5 IVI of aflibercept and not in the control eye of patients treated by ranibizumab. However, the size of these subgroups was very small and does not allow conclusions. However, this would be consistent with the potential systemic diffusion profiles of both anti-VEGF.

To the best of our knowledge, our study is the first to compare the effect of aflibercept to that of ranibizumab on the CT in DME. The strengths of our study are its prospective design with administration of randomized treatments and the use of a control group composed of the contralateral eye of the patients. The main limitation of our study is its small sample size, which is due to the difficulty of recruiting patients with unilateral DME and to the high number of patients lost to follow-up. Studying patients with a longer follow-up would be interesting, but the therapeutic regimens can vary depending on the molecules used and from one patient to another, so that a comparison with an identical number of IVI in all patients would not be possible. The measurements of the choroid are influenced by the nycthemeron, so that they must be made at the same time of the nycthemeron. That is why all our measurements were performed in the afternoon at the clinic between 2 and 5 pm. Another limitation is that patients were included with unilateral DME. Since diabetes is a systemic disease, DME is commonly bilateral. Hence, there is a risk that patients with asymmetric DME have another additional underlying pathology in the affected eye. However, we have rule out the presence of another retinal disease by performing a systematic fluorescein angiography before inclusion.

Moreover, the mean CSCT at baseline seemed different between groups, 206.9 *μ*m in the aflibercept group and 267.5 *μ*m in the ranibizumab group; however, after the Mann–Whitney test, the *p* value was not significant (*p*=0.16). We have no explanation for this difference; however, regarding the medians, we did not find such a difference, 234 *μ*m in the aflibercept group and 270 *μ*m in the ranibizumab group. The higher decrease in the group with the thinner initial CSCT corresponds to a higher percentage of CT reduction, which is consistent with the hypothesis that the CSCT decreases particularly after aflibercept treatment.

## 5. Conclusion

The central CT decreases in DME eyes treated with anti-VEGF IVI in this series, especially in case of treatment with aflibercept.

## Figures and Tables

**Table 1:** Patient characteristics in aflibercept, ranibizumab, and control eyes.

	Aflibercept group (*n* = 13)	Ranibizumab group (*n* = 11)	Control (*n* = 15)
Age (years)	62.1	60	64.7
Type 2 (number)	11	10	15
Baseline BCVA (letters)	64.5	67.1	75.4
Final BCVA (letters)	72.5	79.1	80.5

**Table 2:** Choroidal thickness (CT) and change between M0 and M5 expressed in microns in the different locations of the 3000 *μ*m macular choroidal mapping outside the central 1000 *μ*m in the aflibercept, ranibizumab, and control groups.

	M0	M5	Change	*p*
Aflibercept				
CT sup	204.5	190.5	−14	**0.008**
CT temp	200.5	184.1	−16.4	**0.003**
CT inf	182.9	169. 8	−13.1	**0.03**
CT nasal	190.8	172.9	−17.9	**0.002**
Ranibizumab				
CT sup	270.5	264.7	−5.8	0.19
CT temp	254.2	248.7	−5.5	0.14
CT inf	268.2	254.6	−13.6	0.17
CT nasal	252.2	247.6	−4.6	0.16
Control				
CT sup	240.4	244.3	+3.9	0.86
CT temp	233.5	226.6	−6.8	0.29
CT inf	232	224.9	−7.1	0.13
CT nasal	232.5	232.6	+0.1	0.99

CT sup, superior quadrants; CT temp, temporal quadrants; CT inf, inferior quadrants; CT nasal, nasal quadrants. *p* represents the statistical significance, and the significant values are in bold.

## Data Availability

The data used to support this study are available for one year from the corresponding author upon request.
